# Shifts in Sources of Food but Stable Nutritional Outcomes among Children in the Early Months of the COVID-19 Pandemic

**DOI:** 10.3390/ijerph182312626

**Published:** 2021-11-30

**Authors:** Christine Borger, Courtney Paolicelli, Lorrene Ritchie, Shannon E. Whaley, Jill DeMatteis, Brenda Sun, Thea Palmer Zimmerman, Amanda Reat, Sujata Dixit-Joshi

**Affiliations:** 1 Westat, 1600 Research Boulevard, Rockville, MD 20850, USA; JillDematteis@Westat.com (J.D.); BrendaSun@Westat.com (B.S.); TheaZimmerman@Westat.com (T.P.Z.); SujataDixit-Joshi@westat.com (S.D.-J.); 2 Office of Policy Support, Food and Nutrition Service, US Department of Agriculture, 1320 Braddock Place, Alexandria, VA 22314, USA; Courtney.Paolicelli2@usda.gov (C.P.); Amanda.Reat@usda.gov (A.R.); 3 Nutrition Policy Institute, Division of Agriculture and Natural Resources, University of California, Oakland, CA 94607, USA; lritchie@ucanr.edu; 4 Public Health Foundation Enterprises WIC, Irwindale, CA 91706, USA; Shannon@phfewic.org

**Keywords:** COVID emergency food response, sources of food, school meals, low-income children’s nutrition, food security, longitudinal study

## Abstract

Early in the COVID-19 pandemic, the U.S. Department of Agriculture (USDA), State governments, and school districts took unprecedented steps to mitigate the pandemic’s impact on students’ nutrition. To examine the effect of emergency responses on 6-year-old children’s nutritional outcomes, this study analyzed longitudinal data from a national study of children’s feeding practices, the Special Supplemental Nutrition Program for Women, Infants, and Children—Infant and Toddler Feeding Practices Study-2 (WIC ITFPS-2). Findings include no differences in food insecurity prevalence; however, there were shifts in sources of food, with children in the post-COVID-emergency-declaration (post-ED) group consuming more dietary energy from stores and community food programs and less from restaurants and schools than children in the pre-COVID-emergency-declaration (pre-ED) group (*p* < 0.01 for all comparisons). Examination of within-person mean differences in 2015 Healthy Eating Index scores and nutrient intakes between ages 5 and 6 years revealed few statistically significant differences between the two groups: children in the post-ED group consumed slightly fewer vegetables (*p* = 0.02) and less sodium (*p* = 0.01) than their pre-ED peers. Findings suggest emergency efforts to maintain children’s nutrition were largely successful in the early months of the pandemic. Research is needed to understand the mechanisms by which emergency efforts contributed to these findings.

## 1. Introduction

On 13 March 2020, U.S. President Donald J. Trump declared a national emergency in response to the deepening COVID-19 health crisis [1]. In the weeks that followed, many schools across the U.S. closed their physical doors and transitioned to virtual learning in attempt to curb the spread of the virus. The widespread closure of schools not only upended traditional modes of learning, but it also disrupted the normal distribution of school meals to U.S. students, including children in families with low incomes. With over 29 million students in low-income families participating in school-based meal programs every day, innovative approaches to school meal distributions were urgently needed [2].

The U.S. Department of Agriculture (USDA) funds meal distribution to elementary, middle, and high school students through a number of programs, including the National School Lunch Program (NSLP), the School Breakfast Program (SBP), and the Summer Food Service Program (SFSP). The NSLP is the nation’s second-largest nutrition assistance program [3], and historically, schools provided meals in congregate settings. However, national waivers from the USDA [4] allowed for new approaches to food distribution as an emergency response to the pandemic. Accordingly, many school districts implemented novel methods to deliver food to students in socially distanced ways, including the provision of food items for consumption at home, and drive-through and walk-up centralized pick-up locations. Moreover, new legislation allowed for emergency benefits under the Supplemental Nutritional Assistance Program (SNAP), the nation’s largest nutrition assistance program [5]. In addition, a new nutrition benefit named Pandemic Electronic Benefit Transfer (P-EBT) was created to help families replace the nutrition lost and expense incurred when free and reduced-price meals were no longer available because of school closures [6].

During the early months following the emergency declaration (ED), the USDA also continued to fund a national, longitudinal study of children who were previously enrolled in the Special Supplemental Nutrition Program for Women, Infants, and Children (WIC), the WIC Infant and Toddler Feeding Practices Study-2 (WIC ITFPS-2). WIC is a nutrition assistance program tailored for pregnant and postpartum women and children up to age 5 who are at nutritional risk and whose families have low incomes. WIC ITFPS-2 had been following a cohort of about 3800 children since around the time of their births in 2013 and 2014. Between April 2019 and August 2020, the study children turned 6 years old and were reassessed.

For families in the United States that have low financial resources, a child’s sixth year of life may introduce new feeding challenges. If the child’s birthday meets school age requirements, the child may be attending school and may be receiving free or reduced-price school meals. However, if the child’s birthday does not meet school age requirements, the child may not be attending school when he or she turns 6 and, therefore, may not be participating in federally funded school-based meals programs. If the child is not enrolled in a qualified childcare program, the child may not receive free meals from their childcare provider either. Because the child is no longer categorically eligible for WIC after turning 5 years old, there is the possibility of a gap in nutrition assistance, which may place young children in families with limited resources at risk of food insecurity. Food insecurity, defined by USDA as “the limited or uncertain availability of nutritionally adequate and safe food, or limited or uncertain ability to acquire acceptable foods in socially acceptable ways” [7], is a major public health concern given its association with poor dietary intake among children [8].

WIC ITFPS-2 regularly collects dietary intake data on study children, including around their fifth and sixth birthdays. The sixth-year data provide valuable information on child nutrition and household food security status in the period after the window for WIC eligibility closes. In addition, because sixth-year data collection continued after the COVID-19 ED, the data offer insight into the influence of the pandemic response on children’s food intakes and nutritional outcomes during the initial months of the national emergency.

When examining children’s dietary intakes, both diet quality and consumption of nutrients of public health concern are primary outcomes of interest. The 2015 Healthy Eating Index (HEI-2015) scores facilitate assessment of diet quality because they measure alignment of diets with the Dietary Guidelines for Americans (DGA). From a different perspective, nutrients of public health concern are those that are typically over- or underconsumed by Americans. Research indicates that increasing intakes of underconsumed nutrients of public health concern and limiting intakes of overconsumed nutrients reduce the risk of chronic disease later in life [9,10,11,12,13,14,15,16,17].

Using nationally representative data from WIC ITFPS-2, this study creates two groups of children—those who took their 6-year interview before the COVID-19 ED and those who took their 6-year interview in the early months after the COVID-19 ED—and assesses differences in the groups’ dietary intakes. These two groups of children are referred to as temporal groups because their membership is determined by the date of their 72-month WIC ITFPS-2 interview relative to the time of the ED. The subsequent analysis describes the two groups’ sources of food, HEI-2015 scores, intakes of nutrients of public health concern, and daily total energy. Because young children’s dietary intakes change as they grow, the analytical approach focuses on statistical differences in group means of intrapersonal dietary changes between children’s fifth and sixth years; however, overall means at children aged 6 years for the two groups are also discussed.

## 2. Materials and Methods

WIC ITFPS-2 is a prospective cohort study following children who enrolled in WIC around the time of birth through age 9 years (contract number AG-3198-B11-0020). Enrolled caregivers report on the study children at ages 1, 3, 5, 7, 9, 11, 15, 18, 24, 32, 36, 42, 54, 60, and 72 months, with a planned follow-up at child aged 9 years. Because women could enroll in the WIC ITFPS-2 prenatally or postnatally, each interview is in the field for approximately 15 months. Harrison et al. [18] describe the original protocol for the study. However, because the study has been extended several times since that publication, additional information is available from annual reports [19,20,21]. The Westat Institutional Review Board (IRB) approved WIC ITFPS-2. State and local IRBs approved local study activities as required by local policy.

This research involves secondary analysis of data collected at child aged 60 months and 72 months, referred to as the 60- and 72-month interviews. The interview window for both these interviews is a 6-week window around the study child’s birthday. Data collection for the 60-month interview spans April 2018 through August 2019, while data collection for the 72-month interview spans April 2019 through August 2020.

### 2.1. Participants

WIC ITFPS-2 participants were enrolled in person at study-eligible WIC sites between July and November of 2013. To be eligible for WIC ITFPS-2, a mother had to speak English or Spanish, be at least 16 years old, and be enrolling in WIC for the first time for her current pregnancy or her newborn infant at the time of study enrollment. Mothers who postnatally enrolled in WIC were eligible for the study if, in addition to maternal requirements, their infant was less than 2.5 months old at the time of study enrollment. Mother–child dyads remained in the study regardless of whether they continued with WIC.

### 2.2. Measures and Procedures

Trained telephone interviewers conducted both the 60- and 72-month interviews. At each interview, mothers provided information on sociodemographic characteristics, including participation in school meal programs and WIC; family feeding practices; and other health-related behaviors. Both interviews also included the USDA six-item household food security module, which enquired about food shortage in the prior 12 months [22].

Using the USDA’s scoring algorithm, household food security status was categorized as high/marginal, low, or very low. This study uses the USDA definitions of low and very low food security. Households experiencing low food security “report reduced diet quality and variety but few, if any, indications of reduced food intake,” while households experiencing very low food security “report multiple indications of reduced food intake and disrupted eating patterns, such as skipping meals” [7]. In this analysis, households with either very low or low food security, as assessed by the USDA six-item household food security survey questions about the prior 12 months, qualify as food-insecure households.

As part of the 60- and 72-month interviews, caregivers provided 24-h dietary recall information on their children which was elicited using the USDA Automated Multiple Pass Method (AMPM) [23]. This study uses data from a single dietary recall collected from all participants to assess dietary outcomes including HEI-2015 [24], though a second dietary recall was collected from a 10 percent subsample of WIC ITFPS-2 participants. Findings reflect group-level intakes on a given day.

HEI-2015 is the third and current version of the HEI, a widely recognized tool for assessing how closely food intakes align with the DGA [25]. An HEI-2015 total score indicates how closely an overall diet aligns with the 2015–2020 DGA, while the HEI-2015 component scores assess how well intake meets the specific recommendations within the USDA healthy meal pattern [26]. HEI-2015 total scores range from 0 to 100, with higher scores indicating better alignment with the 2015–2020 DGA. Component scores for total fruits, whole fruits, total vegetables, greens and beans, total protein foods, and seafood and plant proteins range from 0 to 5. Components scores for whole grains, dairy, fatty acids ratio (the ratio of monounsaturated and polyunsaturated fatty acids to saturated fatty acids), refined grains, sodium, added sugars, and saturated fats range from 0 to 10. In addition to HEI-2015 scores, we analyzed daily total energy and seven nutrients of public health concern: fiber, calcium, vitamin D, potassium, added sugars, sodium, and saturated fat [27].

For all dietary intake information, the reported foods, beverages, and dietary supplements were coded using the USDA Food and Nutrient Database for Dietary Studies (FNDDS) to estimate intakes of total energy and 64 nutrients [28]. The USDA Food Pattern Equivalent Database (FPED) (2009–2010 version) was used to estimate intake of 37 food groups. When recalling dietary information, caregivers also reported sources of foods consumed. To facilitate presentation, we recategorized the 25 different sources of food represented in the data into six mutually exclusive groups that encompass all sources: store, restaurant, school, childcare, community food program, and unknown (Supplementary Table S1). We examined changes between 60 and 72 months in the percentage of children’s total dietary energy from each of the six sources.

In both the pre- and post-ED periods, if the respondent indicated receipt of food from a school meal program such as the NSLP, interviewers coded the source as “school” regardless of whether the meal was distributed from a school cafeteria or eaten in one. In both periods, if indicated, interviewers categorized receipt of food directly from WIC as “community food program.” However, for food purchased from stores using WIC, SNAP, or P-EBT benefits, the respondent likely indicated “store” when asked about the source of the food, as these benefits are redeemed primarily only at stores.

### 2.3. Temporal Groups

To examine the impact of responses to the COVID-19 ED on food intakes and nutritional outcomes, two temporal groups were created based on the timing of children’s 72-month interview relative to the ED: a sample of children whose 72-month interviews were conducted prior to the ED (i.e., pre-ED), and a sample of those whose interviews were conducted after the ED (i.e., post-ED). Data from both samples were weighted to produce national estimates. Because regional responses to the pandemic varied greatly during March 2020, we excluded data from interviews conducted between 7 March and 21 March from the two groups to facilitate distinguishing the impact of the emergency response. Consequently, the pre-ED period used data from 30 April 2019 through 6 March 2020 (unweighted *n* = 1560; weighted *n* = 338,589), and the post-ED period used data from 22 March 2020 through 29 June 2020, the last date on which AMPM data were collected (unweighted *n* = 434; weighted *n* = 70,266). Just over one-quarter (27%) of the post-ED interviews were conducted in March 2020. About half (52%) of the post-ED interviews were conducted in April 2020, and about one-fifth (20%) were conducted in May and June 2020.

The study design did not originally envision temporal analysis of a single interview month. Accordingly, we undertook preliminary analyses to ensure that the temporal groups were not systematically different and that the group we planned to exclude from subsequent analyses (unweighted *n* = 143; weighted *n* = 32,372) was not systematically different from the two temporal groups that we planned to compare. We compared estimates for three groups: those who completed the 72-month interview before 7 March (the pre-ED group), those who completed the 72-month interview between 7 March and 21 March (the excluded group), and those who completed the 72-month interview after 21 March (the post-ED group). To ensure that dietary changes were not associated with underlying differences in these three groups, we compared the groups on several sociodemographic characteristics: mother’s educational attainment at child age 54 months; mother’s employment status at child age 54 months and at 72 months; maternal race, ethnicity, marital status, and self-reported weight status at child age 72 months; and household income relative to Federal poverty guidelines, food security status, and participation in nutrition assistance programs at child age 72 months. These sociodemographic characteristics were chosen because prior work with the WIC ITFPS-2 data has shown that they are associated with various aspects of children’s dietary intake [19,20,21]. We used the 72-month interview weights and tested for significance using a second-order Rao–Scott-adjusted chi-square test. We found no evidence to suggest fundamental differences in the three groups other than the employment changes observed. This preliminary work supported analyses involving comparisons of the pre- and post-ED groups.

### 2.4. Statistical Approach

Descriptive analyses presented include means and percentages to summarize characteristics of the pre- and post-ED groups. Because children’s food intakes change as they grow, we examined mean within-person differences between 60- and 72-month intakes for the pre- and post-ED groups when analyzing sources of food, HEI scores, total dietary energy intake, and nutrients of public health concern. To maintain the individual’s correlation in intakes when assessing ratios, these analyses relied on ratios of intakes at the individual level (e.g., person i’s intake of total vegetables divided by person i’s intake of energy). Subsequently, we examined dietary outcomes at 72 months, comparing levels of intakes for the two groups resulting from the changes in intakes between ages 5 and 6. In these analyses, the population ratio method was used [29].

All of the descriptive analyses presented used weighted cases, with the statistical weights accounting for sample selection and nonresponse bias. Weighted findings reflect the entire study-eligible population temporally divided by whether their sixth-year interview occurred pre- or post-ED. There was a 97 percent overlap in participants reporting at both 60 and 72 months, so we used the 72-month cross-sectional sample statistical weights.

SAS statistical software package version 9.4 (SAS Institute, 2013, Cary, NC, USA) was used for data analyses. All statistical tests appropriately accounted for the complex survey design employed in WIC ITFPS-2. All *t*-tests were two-tailed unless otherwise indicated. Statistical significance was at the level of *p* < 0.05.

## 3. Results

Table 1 presents select sociodemographic characteristics of all study participants at 60 and 72 months, as well as characteristics by subsample, for the pre-ED and post-ED groups. Though the focus of this study is on the pre- and post-ED groups, there were some statistically significant changes in the WIC ITFPS-2 population between 60 and 72 months that provide context for more detailed analyses of the two groups at 72 months. A slightly lower percentage of households had 2–3 persons (*p* < 0.01) and a slightly higher percentage had 6 or more persons (*p* = 0.02) at 72 months than at 60 months. As study children aged out of WIC and into formal schooling between the interviews, there was a large jump, nearly 15 percent, in school meal participation (*p* < 0.01) and a significant drop, nearly 20 percent, in household WIC participation (*p* < 0.01). At 72 months, a lower percentage had household incomes at or below 75 percent of the Federal poverty guidelines (*p* = 0.02) and a higher percentage had incomes above 130 percent of the guidelines (*p* = 0.01) than at 60 months. Concomitantly, the percentages of families reporting high or marginal household food security was higher at 72 months than at 60 months (*p* < 0.01), while the percentage of households reporting low or very low food security (combined) was lower (*p* < 0.01).

Examination of the characteristics of the pre- and post-ED groups at 72 months revealed that full-time maternal employment was 10 percentage points lower and maternal unemployment was 10 percentage points higher in the post-ED group than in the pre-ED group. In both cases, the difference was statistically significant (*p* < 0.01). The percentage of households reporting incomes less than 75 percent of the Federal poverty guidelines also significantly differed between groups (*p* = 0.04), with a larger percentage of those in the post-ED group than in the pre-ED group reporting incomes below this level. Participation in the school meal programs (NSLP, SBP, and/or SFSP) was more prevalent in the post-ED group than the pre-ED (*p* = 0.05), as was participation in WIC by mothers (*p* = 0.05) and infants (*p* = 0.04).

Table 2 presents the mean within-person differences in energy intake between 60 and 72 months by food source for the pre- and post-ED groups. Negative mean differences indicate that 60-month values were more frequently higher than the 72-month values, while positive mean differences indicate that the 72-month values were more frequently higher than the 60-month values. Between child ages 60 and 72 months, children in the pre-ED group generally obtained less dietary energy from stores while children in the post-ED group generally obtained more (*p* < 0.01). The opposite occurred when examining food obtained from restaurants and schools. Six-year-olds interviewed pre-ED generally obtained more dietary energy from restaurants and schools, while 6-year-olds interviewed post-ED obtained less (*p* < 0.01 for both comparisons). Children in the pre-ED group also generally obtained less dietary energy from community food programs while children in the post-ED group obtained more (*p* < 0.01). Energy obtained from childcare declined for both groups, on average, but the comparison of mean differences was not statistically significant between pre- and post-ED groups.

Table 3 presents the mean within-person differences in the total and component HEI-2015 scores, intakes on a given day of seven nutrients of public health concern, and total daily energy for the pre- and post-ED groups. Mean differences for HEI-2015 total scores and 11 of 13 component scores were not significantly different for the two groups. The difference in mean HEI-2015 total vegetable component scores was significant (*p* < 0.02), with the post-ED group exhibiting a small deterioration in scores, on average, and a small improvement in scores for the pre-ED group. The difference in mean HEI-2015 sodium component scores was also significant (*p* = 0.01), with the post-ED exhibiting an improvement in scores between 60 and 72 months, on average, and the pre-ED group exhibiting a small deterioration. There were no statistically significant differences between groups in intakes on a given day in the seven nutrients of public health concern; however, there was a significant difference in daily total energy intake on a given day (*p* = 0.02), with the post-ED group exhibiting a larger mean difference than the pre-ED group.

Analyses of within-person differences may account for individual trajectories between the fifth and sixth years; however, levels of intakes at 72 months provide important indicators of diet quality after these changes. To assess whether the nutritional intakes of the pre- and post-ED groups were similar, we compared mean HEI scores and nutrient intakes using the population ratio method. As mentioned, because the pre- versus post-ED classification was not randomly assigned (but based on the timing of the 72-month interview), we first examined characteristics of the two groups to ensure there were no systematic differences that might confound the results of the HEI score comparison; other than employment status (which is to be expected, as a result of immediate effects of the ED), no differences were found.

There were no significant differences between the pre- and post-ED groups for the majority of HEI scores and nutrients analyzed (Table 4). There were significant differences in mean HEI scores for total vegetables (*p* = 0.05), with the post-ED group scoring slightly lower than the pre-ED group, and in mean HEI sodium component scores (*p* < 0.01), with the post-ED group scoring slightly higher than the pre-ED group, where higher HEI sodium component scores indicated lower sodium intake given energy. However, this analytic approach also indicated significant differences in intakes of refined grains (*p* = 0.03) and total saturated fat (*p* = 0.04), with the post-ED group exhibiting higher scores for refined grains and lower scores for saturated fats than the pre-ED group. Among the seven nutrients of public health concern considered for comparison, significant differences were identified in vitamin D intakes, both in total grams (*p* < 0.01) and in grams per 1000 kilocalories (*p* < 0.01), with the post-ED group exhibiting higher intakes than the pre-ED group in both cases. Additionally, a significant difference was identified in intakes of saturated fat as a percentage of dietary energy (*p* = 0.01), with the post-ED group consuming a slightly larger percentage than the pre-ED group.

## 4. Discussion

This study used data from the WIC ITFPS-2 to examine changes in dietary intakes prior to and after the COVID-19 health ED. Two temporal groups, a pre-ED group and a post-ED group, were compared on many sociodemographic characteristics to determine whether the approach was feasible. Based on data from the 72-month interview, only maternal employment and household income differed significantly. These were interpreted as effects of the pandemic rather than an underlying difference in the groups because it is well established that employment in the United States initially fell dramatically in response to COVID-19 mitigation strategies [30].

Using the two groups identified, there were shifts in children’s sources of dietary energy after the 13 March 2020 health ED: a greater share of children’s dietary energy came from stores and community food programs, and less from restaurants and schools, in the post-ED period than in the pre-ED period. This finding aligns with work by Murphy et al. (2020). Using a cross-continental sample of adults from Ireland, Great Britain, the United States, and New Zealand, they found that all four regions had reductions in food from restaurants (i.e., takeaways) because of the pandemic [31].

Examination of children’s within-person changes in diet quality measures revealed that, compared to the pre-ED group, the post-ED group exhibited a slight decline in vegetable consumption and a small improvement in sodium consumption between 60 and 72 months. The finding for vegetable consumption is noteworthy given that previous literature suggests school meals tend to include more vegetables than those served at home [32]. The findings regarding lower sodium intake by the post-ED group than the pre-ED group are consistent with reductions in food from restaurants, which tend to offer foods higher in sodium than foods eaten at home or schools [33]. Examination of mean within-person changes for energy and nutrients of public health concern indicated that the post-ED group exhibited a larger increase in daily total dietary energy than the pre-ED group.

Viewed in light of reduced prevalence of food insecurity at child aged 72 months compared to child aged 60 months, it is, perhaps, unsurprising that there were few significant differences between the pre- and post-ED groups in dietary outcomes between 60 and 72 months. Examination of within-person changes found that mean HEI total scores were similar between the groups, as were 11 of 13 component scores and all of the nutrients analyzed.

The post-ED group had a much larger mean difference in dietary energy intake between 60 to 72 months than the pre-ED group (Table 3); however, mean daily total energy intake did not differ significantly between the groups at age 72 months (Table 4). This suggests that the children who turned 5 later in the study may have started slightly behind their older peers and caught up to them at age 6. The current study could not assess the adequacy of energy intake relative to energy expenditure; nonetheless, it is reassuring that, based on population ratio methodology, intakes were similar across both groups.

Analysis of levels of intake at child aged 72 months indicated some statistically significant differences between the pre- and post-ED groups; however, magnitudes of differences were generally small. The differences found in nutrient intakes may be related to the types and/or amounts of foods consumed, and future research may want to examine specific foods consumed.

One of the key findings from this study is that despite having a lower prevalence of maternal full-time employment and a higher prevalence of the lowest-income households within the post-ED group at 72 months, overall, a lower percentage of families represented in the longitudinal WIC ITFPS-2 data reported low or very low food security at 72 months than at 60 months. This finding contrasts with findings of increased food insecurity in the early months of the COVID-19 national emergency, particularly in households with children [34,35,36]; however, the finding aligns with recent research finding that the prevalence of food insecurity did not change between 2019 and 2020 [37]. In this context, it is important to remember that the USDA six-item household food security instrument used in this study inquires about the past 12 months, so much of the time period covered was prior to the ED.

The results of this study suggest that, even with all of the hardships many families experienced at the onset of the pandemic, a high percentage of low-income 6-year-old children were able to access a similar quantity of dietary energy in the post-ED period as their pre-ED peers, with minimal differences in diet quality. Federal, State, and local emergency response efforts enacted in spring 2020 likely helped facilitate this, at least partially. Additional research is needed to understand the extent to which each of these efforts contributed to the decline in prevalence of food insecurity among low-income families with past exposure to Federal nutrition assistance programs, particularly WIC.

The increases in the percentages of WIC ITFPS-2 families reporting participation in school meal programs and/or WIC between the pre- and post-ED groups further suggest that prior WIC participation for the study child may have been a protective factor as levels of unemployment rose, providing actionable knowledge on the process for receiving nutrition assistance through programs designed to serve families with children. In this context, we reiterate that the dietary recall data extended through the end of June 2020; therefore, the prevalence estimates for food insecurity are for the early months of the pandemic response. As the national health emergency continued, the prevalence of food insecurity in this vulnerable population may have changed notably.

There are three notable limitations to this research. The first is that the study data do not permit exploration of how families acquired food and beverages. WIC ITFPS-2 did not collect financial information other than household income. As full-time employment fell, it is possible that study participants dipped into financial reserves. Additionally, research examining coping strategies suggests that participants may have tapped relationship assets [38]. Unemployment benefits, expanded SNAP and P-EBT benefits, and efforts to distribute produce, nonperishable foods, and milk through schools and community programs may also have facilitated food acquisitions. WIC ITFPS-2 did not specifically address these coping strategies, and additional studies are needed to understand the role of these sources of support in food security and diet quality of children in families with low incomes.

A second limitation is that while the AMPM is used extensively to assess dietary intake, it is subject to recall error. This is particularly important when considering the sources of food. For example, between the pre- and post-ED periods, federally funded school meals changed from service and consumption at school sites to distribution at schools and other sites for consumption outside of schools—primarily at home. In the post-ED period, once these distributed foods were in the home, it may have been challenging for respondents to identify exactly which foods or beverages came from school distributions and which were purchased at stores. This may have resulted in underreporting of foods from schools as a source and overreporting of foods from stores or community food programs.

A third limitation is the inability to state whether changes in daily energy intakes were appropriate for the children. WIC ITFPS-2 does not collect detailed information on children’s physical activity levels or weight at the time of diet intake measurement; thus, it is not possible to assess energy intakes in relation to the child’s activity or requirements. Though the within-person findings indicate a larger change in daily total energy intake for the post-ED group than the pre-ED group, the changes left both groups at similar mean levels when the children were 6 years old.

## 5. Conclusions

The results of this study highlight the likely protective effect of Federal, State, and local government efforts on household food security during the early months of the pandemic. Using data from WIC ITFPS-2, this study found that, nationally, about 25 percent of households with 5-year-old children who had previously enrolled in WIC around the time of birth reported low or very low food security between April 2018 and August 2019, while only 20 percent of these households reported low or very low food security when the children were 6 years old, between April 2019 and August 2020. Analyses of household food security by whether the study child turned 6 years old prior to or after the COVID-19 ED (March 2020) indicated no difference in the percentages reporting low or very low food security (20.6% compared to 20.2%), even though maternal full-time employment was notably higher in the pre-ED period than the post-ED period (40.0% compared to 29.9%), based on pairwise *t*-tests.

Consistent with the national emphasis on social distancing, which closed many businesses and schools, more of children’s dietary energy was consumed at home. Analysis revealed statistically significant differences in sources of dietary energy between ages 5 and 6 years for two groups of children—those who turned 6 years old before the U.S. COVID-19 ED and children who turned 6 years old after the ED. Pairwise *t*-tests of mean intrapersonal differences for the two groups indicated families relied more heavily on stores (−3.56% vs. 8.51%) and community food programs (−0.49% vs. 1.26%) as sources for children’s dietary energy, and less heavily on restaurants (0.98% vs. −5.24%) and schools (4.10% vs. −3.59%) in the post-ED period than in the pre-ED period.

There were few statistically significant differences in mean dietary intakes of 6-year-olds assessed pre-ED compared to those assessed post-ED, and overall mean diet quality scores and energy intakes were similar based on *t*-tests. Future studies should not only continue to examine the ongoing impacts of the pandemic on food insecurity and diet quality of low-income families in the United States but should also focus on the mechanisms by which Federal, State, and local efforts may mitigate food insecurity.

## Figures and Tables

**Table 1:** Select sociodemographic characteristics of respondents during the children’s fifth and sixth years.

Sociodemographic C0 Haracteristic	Fifth-Year ^a^ Interview	Sixth-Year ^b^ Interview			*p*-Value from Comparison of Pre/Post-ED Groups
	Nationally Representative Group % ^c^ (SE)	Nationally Representative Group % ^c^ (SE)	Pre-ED ^d^ Group % ^c^ (SE)	Post-ED ^e^ Group % ^c^ (SE)	
Caregiver’s Employment Status ^f^					
Working full-time for pay	35.6 (1.4)	38.9 (1.9)	40.0 (2.2)	29.9 (2.2)	<0.01 *
Working part time for pay	20.7 (1.2)	20.4 (1.1)	20.9 (1.4)	21.1 (1.9)	0.93
Not working	43.7 (1.5)	40.7 (1.8)	39.2 (1.9)	49.0 (2.7)	<0.01 *
Unweighted *n* ^g^	2560	2137	1560	434	
Weighted *n* ^g^	440,806	441,226	338,589	70,266	
Household Size					
2–3 people	28.7 (1.6)	25.8 (1.7)	26.7 (1.9)	22.9 (2.6)	0.23
4–5 people	53.9 (1.4)	54.7 (1.4)	54.6 (1.8)	53.6 (2.4)	0.74
6 or more people	17.3 (1.2)	19.5 (1.3)	18.7 (1.3)	23.6 (3.0)	0.13
Household Income Relative to Federal Poverty Guidelines					
75% or below	40.9 (1.6)	37.7 (1.6)	36.7 (1.6)	42.8 (2.9)	0.04 *
Above 75% but no more than 130%	31.1 (1.2)	30.0 (1.6)	30.5 (1.9)	27.3 (2.4)	0.37
Above 130%	28.0 (1.6)	32.3 (1.8)	32.7 (2.1)	29.9 (2.7)	0.38
At or below 200%	89.9 (0.9)	88.1 (1.1)	87.6 (1.4)	91.7 (1.9)	0.05
Household Participation in Federal Nutrition Programs					
Supplemental Nutrition Assistance Program (SNAP)	42.7 (1.6)	41.2 (1.5)	41.3 (1.6)	40.6 (2.9)	0.81
National School Lunch Program (NSLP), School Breakfast Program (SBP), and/or Summer Food Service Program (SFSP)	51.9 (2.0)	64.6 (2.1)	63.0 (2.1)	70.4 (3.8)	0.05 *
Special Supplemental Nutrition Program for Women, Infants, and Children (WIC)	44.7 (2.3)	25.7 (1.4)	23.8 (1.6)	29.0 (3.0)	0.13
WIC for self (pregnant/postpartum mother) ^h^	N/A	8.2 (0.8)	6.7 (0.9)	10.6 (1.7)	0.05 *
WIC for infants under 12 months old ^h^	N/A	8.7 (0.9)	7.7 (1.0)	11.9 (1.8)	0.04 *
WIC for children ages 1–5 years old ^h^	N/A	20.7 (1.2)	19.0 (1.3)	23.4 (2.6)	0.10
Participate in any of the three Federal nutrition programs assessed (SNAP, NSLP, SBP, SFSP, and/or WIC)	77.6 (1.6)	77.0 (1.5)	75.9 (1.6)	80.4 (2.6)	0.11
Household Food Security Status					
High or marginal	75.1 (1.1)	79.7 (1.1)	79.3 (1.2)	79.8 (2.4)	0.87
Low	14.3 (0.8)	12.2 (0.8)	12.5 (1.0)	11.6 (2.2)	0.72
Very low	10.6 (0.8)	8.1 (0.6)	8.1 (0.8)	8.6 (1.6)	0.81
Unweighted *n* ^i^	2526	2137	1560	434	
Weighted *n* ^i^	440,770	441,226	338,589	70,266	

^a^ 60-month interview data unless otherwise noted. ^b^ 72-month interview data. ^c^ Percentages may not sum to 100 percent due to rounding. ^d^ Pre-emergency declaration (pre-ED) data are from 30 April 2019 through 6 March 2020. ^e^ Post-emergency declaration (post-ED) data are from 22 March 2020 through 29 June 2020. ^f^ Employment status was not assessed at the 60-month interview, so the fifth-year data are from the 54-month interview. ^g^ *n* is the number of respondents to the 54- and 72-month interviews. ^h^ Not assessed at the 54- or 60-month interview. N/A indicates that the data are not available. ^i^ *n* is the number of respondents who completed the 60- and the 72-month interviews. * Bolded indicates statistically significant pairwise difference between pre-/post-ED values at *p* < 0.05 based on two-tailed *t*-test. Two-digit presentation meant some significant *p*-values were rounded to 0.05.

**Table 2:** Mean within-person differences between 60- and 72-month sources of dietary energy as a percentage of total energy for pre-emergency-declaration (pre-ED) and post-emergency-declaration (post-ED) groups.

Food Source	Pre-ED Group Mean Difference in Percentage of Total Energy (72-Month Value Minus 60-Month Value)	Post-ED Group Mean Difference in Percentage of Total Energy (72-Month Value Minus 60-Month Value)	*p*-Value from Comparison of Pre/Post-ED Group Mean Differences
Store ^a^	−3.56	8.51	<0.01 *
Restaurant	0.98	−5.24	<0.01 *
School	4.10	−3.59	<0.01 *
Childcare	−0.94	−0.70	0.51
Community food program	−0.49	1.26	<0.01 *
Unknown source	−0.10	−0.24	0.77

^a^ Store includes supermarket, grocery store, warehouse store, commissary, convenience store, drug store, gas station, specialty store-bakery, bagel, coffee, deli, doughnut, seafood, ethnic food, health food, liquor, beer, ice cream, dairy, gift shop, company store, food/beverage sample from store, food/beverage from store salad/food bar, deliveries from store, produce stand, farmer’s market, weight loss stores/programs (Weight Watchers, Jenny Craig, LA Weight Loss Center). Also included for these analyses are foods grown or caught by someone you know and fish from the ocean. * Bolded indicates a statistically significant pairwise difference in mean differences pre/post-ED at *p* < 0.05.

**Table 3:** Mean within-person differences between the 60- and 72-month interviews for Healthy Eating Index (HEI-2015) total and component scores ^a^, energy and macronutrients, and nutrients of public health concern for the pre-emergency-declaration (pre-ED) and post-emergency-declaration (post-ED) groups.

HEI-2015 and Dietary Component	Pre-ED Group Mean Difference (72 Month–60 Month)	Post-ED Group Mean Difference (72 Month–60 Month)	*p*-Value from Comparison of Pre/Post-ED Group Mean Differences
HEI-2015 Score			
Total	−2.3	−1.8	0.50
Total vegetables	0.1	−0.2	0.01 *
Total greens and beans	0.1	−0.1	0.13
Total fruit	−0.2	0.0	0.18
Total whole fruit	−0.2	0.0	0.14
Total whole grains	−0.6	0.0	0.13
Total dairy	−0.2	−0.2	0.99
Total protein foods	0.0	0.1	0.68
Seafood and plant protein	0.0	−0.1	0.55
Fatty acid ratio	−0.2	−0.5	0.25
Sodium	−0.4	0.5	<0.01 *
Refined grains	−0.4	−0.3	0.57
Saturated fat	−0.2	−0.6	0.10
Added sugars	−0.3	−0.3	0.78
Nutrients of Public Health Concern			
Fiber, total dietary (g) intake	0.4	1.6	0.07
Fiber, total intake by energy intake (g/1000 kcal)	−0.3	0.1	0.23
Calcium (mg) intake	24.8	93.7	0.13
Calcium, total intake by energy intake (mg/1000 kcal)	−20.5	−19.5	0.97
Vitamin D (D2 + D3) (mcg) intake	−0.4	0.5	0.13
Vitamin D (D2 + D3), total intake by energy intake (mcg/1000 kcal)	−0.6	−0.2	0.28
Potassium (mg) intake	49.2	125.9	0.23
Potassium, total intake by energy intake (mg/1000 kcal)	−45.7	−82.5	0.08
Total added sugars intake (% kcals)	0.6	0.7	0.86
Sodium (mg) intake	158.0	243.3	0.26
Sodium, total intake by energy intake (mg/1000 kcal)	21.3	−31.3	0.12
Total saturated fat intake (% kcals)	−5.9	−5.6	0.33
Total Daily Energy			
Energy (kcal) intake	81.3	191.6	0.01 *

^a^ Total HEI-2015 scores range from 0 to 100 with higher scores indicating greater alignment with the 2015–2020 Dietary Guidelines for Americans (DGA). Component scores for total fruits, whole fruits, total vegetables, greens and beans, total protein foods, and seafood and plant proteins range from 0 to 5. Components scores for whole grains, dairy, fatty acid ratio (the ratio of monounsaturated and polyunsaturated fatty acids to saturated fatty acids), refined grains, sodium, added sugars, and saturated fats range from 0 to 10. * Bolded indicates statistically significant pairwise difference in mean differences pre/post-ED at *p* < 0.05.

**Table 4:** 2015 Healthy Eating Index (HEI-2015) total and component scores ^a^ and nutrients of public health concern and energy, nationally representative, pre-emergency-declaration (pre-ED) and post-emergency-declaration (post-ED) groups.

HEI-2015 and Dietary Component		72-Month Interview			*p*-Value from Comparison of Pre/Post-ED Group Means
		Nationally Representative Group	Pre-ED Group	Post-ED Group	
		Mean (SE)	Mean (SE)	Mean (SE)	
HEI-2015 Score	Maximum Score				
Total	100	52.1 (0.5)	51.9 (0.5)	52.3 (0.6)	0.66
Total vegetables	5	2.3 (0.0)	2.3 (0.0)	2.1 (0.1)	0.05 *
Total greens and beans	5	1.2 (0.1)	1.2 (0.1)	1.7 (0.1)	0.34
Total fruit	5	3.8 (0.0)	3.8 (0.1)	3.9 (0.1)	0.36
Total whole fruit	5	3.4 (0.1)	3.4 (0.1)	3.4 (0.1)	0.89
Total whole grains	10	2.6 (0.1)	2.6 (0.1)	2.9 (0.2)	0.15
Total dairy	10	7.7 (0.1)	7.6 (0.1)	7.5(0.2)	0.58
Total protein foods	5	3.8 (0.0)	3.8 (0.0)	3.9 (0.1)	0.32
Seafood and plant protein	5	0.9 (0.1)	0.9 (0.1)	0.8 (0.1)	0.65
Fatty acid ratio	10	3.2 (0.1)	3.2 (0.1)	3.0 (0.1)	0.21
Sodium	10	4.6 (0.1)	4.4 (0.1)	5.1 (0.2)	<0.01 *
Refined grains	10	5.2 (0.1)	5.1 (0.1)	5.7 (0.2)	0.03 *
Saturated fat	10	5.9 (0.1)	5.9 (0.1)	5.5 (0.2)	0.04 *
Added sugars	10	7.6 (0.1)	7.7 (0.1)	7.4 (0.2)	0.18
Nutrient of Public Health Concern	Recommended Level				
Fiber, total dietary (g) intake	17–20 g/d ^b^	14.8 (0.2)	14.6 (0.2)	15.0 (0.4)	0.43
Fiber, total intake by energy intake (g/1000 kcal)	14 g/1000 kcal ^b^	8.5 (0.1)	8.4 (0.1)	8.4 (0.2)	0.84
Calcium (mg) intake	1000 mg/d ^c^	1075.2 (15.5)	1062.4 (20.2)	1102.7 (32.0)	0.32
Calcium, total intake by energy intake (mg/1000 kcal)	N/A ^d^	614.3 (7.2)	611.4 (8.3)	619.2 (11.7)	0.59
Vitamin D (D2 + D3) (mcg) intake	15 mcg/d ^c^	8.3 (0.2)	8.0 (0.2)	9.3 (0.3)	<0.01 *
Vitamin D, total intake by energy intake (mcg/1000 kcal)	N/A ^d^	4.7 (0.1)	4.6 (0.09)	5.2 (0.19)	<0.01 *
Potassium (mg) intake	2300 mg/d ^e^	2298.1 (19.7)	2283.6 (29.4)	2297.8 (47.8)	0.82
Potassium, total intake by energy intake (mg/1000 kcal)	N/A ^d^	1313.0 (10.2)	1314.2 (10.7)	1290.2 (20.4)	0.30
Total added sugar intake (% kcals)	<10% kcals ^f^	10.5 (0.1)	10.4 (0.2)	11.0 (0.4)	0.18
Sodium (mg) intake	1000 mg/d ^e^	2831.1 (40.0)	2831.6 (53.7)	2802.8 (78.7)	0.77
Sodium, total intake by energy intake (mg/1000 kcal)	N/A ^d^	1617.5 (13.6)	1629.6 (14.4)	1573.8 (25.6)	0.06
Total saturated fat (% kcals)	<10% kcals ^f^	5.0 (0.0)	11.2 (0.1)	11.7 (0.1)	0.01 *
Total Daily Energy					
Energy (kcal) intake	N/A ^d^	1750.3 (19.4)	1737.6 (27.7)	1780.9 (34.3)	0.38

^a^ Total HEI-2015 scores range from 0 to 100 with higher scores indicating greater alignment with the 2015–2020 Dietary Guidelines for Americans (DGA). Component scores for total fruits, whole fruits, total vegetables, greens and beans, total protein foods, and seafood and plant proteins range from 0 to 5. Components scores for whole grains, dairy, fatty acid ratio (the ratio of monounsaturated and polyunsaturated fatty acids to saturated fatty acids), refined grains, sodium, added sugars, and saturated fats range from 0 to 10. ^b^ From the Dietary 2020–2025 Guidelines for Americans (DGA). Recommended level for 4–8-year-old females consuming 1200 kcal/d is 17 g/d; recommended level for 4–8-year-old males consuming 1400 kcal/d is 20 g/d. ^c^ Recommended Daily Allowance (RDA). ^d^ Not available. ^e^ Adequate intake (AI). ^f^ 2020–2025 Dietary Guidelines for Americans (DGA). * Bolded indicates statistically significant pairwise mean difference pre/post-ED at *p* < 0.05.

## Data Availability

Data can be obtained by contacting the U.S. Department of Agriculture Food and Nutrition Service at OPSDataRequests@usda.gov.

## Supplementary Materials

The following are available online at https://www.mdpi.com/article/10.3390/ijerph182312626/s1, Table S1: Aggregated categories for sources of food.
