# Supplementary material for: Shifts in Sources of Food but Stable Nutritional Outcomes among Children in the Early Months of the COVID-19 Pandemic
Source: Int J Environ Res Public Health. 2021 Nov 30;18(23):12626. doi: 10.3390/ijerph182312626 (PMC8656921; doi:10.3390/ijerph182312626)
Supplement: Supplementary file 1 [file ijerph-18-12626-s001.zip › ijerph-1453440-supplementary.pdf]

Table S1. Aggregated categories for sources of food.

Original category	Aggregated category
Store	Store
Mail order purchase	Store
Grown or caught by you or someone you know	Store
Fish from the ocean	Store
Restaurant with waiter/waitress	Restaurant
Restaurant fast, food/pizza	Restaurant
Bar/tavern/lounge	Restaurant
Restaurant no additional info	Restaurant
Cafeteria not at school	Restaurant
Residential dining facility	Restaurant
Sport recreation, or entertainment facility	Restaurant
Street vendor, vending truck	Restaurant
Fundraiser sales	Restaurant
Vending machine	Restaurant
Cafeteria at school	School
Child care center	Childcare
Family/adult day care center	Childcare
Soup kitchen/shelter/food pantry	Food pantry
Community food program, other	Food pantry
Common coffee pot or snack tray	Food pantry
From someone else/gift	Food pantry
Other, Specify	Unknown
